# Altered Resting-State Network in Adolescents with Problematic Internet Use

**DOI:** 10.3390/jcm11195838

**Published:** 2022-10-01

**Authors:** Kristiana Siste, Jacub Pandelaki, Jun Miyata, Naoya Oishi, Kosuke Tsurumi, Hironobu Fujiwara, Toshiya Murai, Martina Wiwie Nasrun, Tjhin Wiguna, Saptawati Bardosono, Rini Sekartini, Riza Sarasvita, Belinda Julivia Murtani, Lee Thung Sen, Karina Kalani Firdaus

**Affiliations:** 1 Department of Psychiatry, Faculty of Medicine, Universitas Indonesia—Dr. Cipto Mangunkusumo National Central General Hospital, Jakarta 10430, Indonesia; 2 Department of Radiology, Faculty of Medicine, Universitas Indonesia—Dr. Cipto Mangunkusumo National Central General Hospital, Jakarta 10430, Indonesia; 3 Department of Psychiatry, Graduate School of Medicine, Kyoto University, Kyoto 606-8501, Japan; 4 Medical Innovation Center, Graduate School of Medicine, Kyoto University, Kyoto 606-8501, Japan; 5 Department of Neuropsychiatry, Graduate School of Medicine, Kyoto University, Kyoto 606-8501, Japan; 6 Decentralized Big Data Team, RIKEN Center for Advanced Intelligence Project, Tokyo 103-0027, Japan; 7 The General Research Division, Osaka University Research Center on Ethical, Legal, and Social Issues, Osaka 565-0871, Japan; 8 Department of Clinical Nutrition, Faculty of Medicine, Universitas Indonesia—Dr. Cipto Mangunkusumo National Central General Hospital, Jakarta 10430, Indonesia; 9 Department of Pediatrics, Faculty of Medicine, Universitas Indonesia—Dr. Cipto Mangunkusumo National Central General Hospital, Jakarta 10430, Indonesia; 10 Faculty of Psychology, Soegijapranata University, Central Java 50234, Indonesia

**Keywords:** resting state, functional connectivity, adolescent, internet addiction, triple-network model, emotional symptoms

## Abstract

Problematic internet use (PIU) is increasingly recognized as a mental health concern, particularly among adolescents. The resting-state functional connectivity (rsFC) of the triple-network model has been described inconsistently in PIU. Using resting-state fMRI (rsFMRI) and hypothesizing a lower rsFC between default mode (DMN) and central executive networks (CEN) but a higher rsFC within the salience network (SN), this study scrutinized the neural substrates of PIU adolescents. A total of 30 adolescents with PIU and 30 control subjects underwent rsFMRI. The severity of PIU was evaluated by the Internet Addiction Test. Additionally, personality traits as well as emotional and behavioral problems were evaluated by the Temperament and Character Inventory (TCI) and the Strength and Difficulties Questionnaire (SDQ), respectively. Focusing on the DMN, SN, and CEN, we compared rsFC values between PIU and the control. Subsequently, within the combined group of subjects, TCI and SDQ correlation and mediation effects were investigated. Higher rsFC values of the left lateral prefrontal cortex (LPFC(L)) with the left anterior insula (aIns(L)) were observed for PIU than for the control, while rsFCs of the LPFC(L) with the medial PFC (MPFC), LPFC(L), as well as with the right lateral parietal cortex (LP(R)) were lower for PIU. Among these significant group differences, the rsFC between the LPFC(L) and MPFC was mediated by emotional symptoms (standardized β = −0.12, 95% CI −0.29, −0.0052). The dysfunctional attention switching and incentive salience regulated by the SN were implicated as being a neural correlate of PIU, and this relationship would in part be explained by the emotional dysregulation associated with PIU in adolescents.

## 1. Introduction

The modern world has presented potential novel disorders, including problematic internet use (PIU). PIU has garnered great interest in the scientific world, with its subtype of the Internet Gaming Disorder (IGD) being recognized as a behavioral addiction requiring further studies in the Diagnostic and Statistical Manual for Mental Disorders, 5th edition and as a Gaming Disorder (GD) in the 11th edition of the International Classification of Diseases. In 2014, the worldwide prevalence of PIU was estimated to be 6% [1], and a more recent review from 2018 revealed a rate of 4.6% for IGD among adolescents [2]. The global rate contrasted with the data in Southeast Asia, which projected a 20.0% and 10.1% pooled prevalence for PIU and GD [3], suggesting a more pressing issue within the region. Notably, studies have indicated a worsening trend of PIU post-COVID-19 pandemic among adolescents and youths [4,5] due to physical isolation and digital transitioning of social activities. Another possibility explaining the variability in PIU prevalence is the difficulties of detecting PIU in adolescents and youths [6], with either DSM-5 or ICD-11 criteria demonstrating discrepancies [7]. Additionally, numerous instruments have been utilized to assess PIU at the population level, of which one of the most commonly used is the Internet Addiction Test (IAT) [1,3].

One of the areas requiring further data is the neurobiological processes underlying PIU, apart from its natural history and diagnostic criteria [8]. In substance use disorder, the dysfunction of the reward system and inhibitory control has been identified as playing a central role [9,10,11]. The deficits were markedly observed among adolescents, exhibiting heightened cognitive impulsivity and compulsive behavior, in part due to the yet developed prefrontal and orbitofrontal cortices [12,13]. However, these neural areas are malleable by not only genetic factors but also environmental variables [14].

Resting-state functional magnetic resonance imaging (rsfMRI) is an increasingly popular method for analyzing neural correlates through scrutinization of the blood-oxygen-level-dependent (BOLD) fluctuations. Several studies have demonstrated the reproducibility of the resting-state functional connectivity (rsFC) elucidated through rs-fMRI [15,16]. The intrinsic connectivity networks (ICNs) revealed by the rsFC have also been proposed to be predictive of behaviors [17] or psychopathology [18,19]. Studies using rsfMRI on PIU patients revealed diminished connectivity between subcortical regions and the PFC and parietal cortex [20], which are the cortices of the central executive network (CEN). In contrast, a positive correlation was observed between the insular region and anterior cingulate cortex (ACC) [21], which represent the salience network and the insula (SN). rsfMRI also allowed the study of the default mode network (DMN), which is deactivated during task performance and activated during rest, is indicated to control thought processes, and is altered in substance [22,23,24] and nonsubstance addiction [25,26,27]. Recently, the “triple-network model” proposed that the CEN, SN, and DMN play key roles in various psychiatric disorders, including addiction [28]. Emerging discoveries pointed to the central role of the SN in stimuli detection, in which the SN disengages the DMN and engages the CEN [28]. In contrast, Zhang et al. demonstrated a higher rsFC of SN-DMN but lower rsFC of the SN-CEN in IGD compared to controls [29]. However, another study demonstrated weaker rsFCs between CEN nodes and the SN-DMN [30], and differences except for a weaker rsFC between CEN nodes in PIU compared to healthy controls [31]. Thus, the literature on the rsFC of PIU is inconsistent.

Temperaments are postulated to be partially heritable and persist throughout the life stages [32]. PIU had been associated with several temperament profiles, such as high novelty seeking (NS), high harm avoidance (HA), and low reward dependence (RD) [33,34,35]. Temperament continuously affects cognition development, adaptive behavior, and executive functioning from infancy through maturation [36,37,38,39] and is predictive of impulsive behavior [40]. Similarly, emotional dysregulation and behavioral problems hampered proper executive regulatory function development, from attention flexibility to working memory [41] and cognitive inhibitory process [42]. Through empirical studies, PIU in adolescents was found to be significantly predicted by impulsivity, mediated through an emotion-focused coping mechanism, for both male and female adolescents [43], and linked with deficits in multiple cognitive functions (from attentional inhibition to impaired decision-making) [44]. The brain changes among PIU adolescents were suggested to be mediated by several factors, including psychological, social, and educational attributes [21].

In this exploratory study, we explicated the functional networks of PIU among a sample of Indonesian adolescents. As past studies have indicated the problem of cognitive inhibition and decreased connectivity of the prefrontal cortical circuits, we hypothesized that adolescents with PIU would present with a lower rsFC in the CEN and DMN compared to the control group. We also expected increased rsFCs of the SN in PIU, because prior evidence indicates heightened insular and ACC rsFC values corresponding to incentive salience. Further, the temperament and psychopathologies variables would mediate the relationship between IAT scores and rsFC modulations.

## 2. Materials and Methods

### 2.1. Subjects

This study involved 30 PIU subjects as the test group and 30 subjects non-PIU as the control group. The subjects were junior and senior high school students and were randomized from a pool of 643 participants recruited through a prior study (see [45] for detailed recruitment process). All subjects’ parents and/or guardians provided written informed consent. The subjects also underwent structured psychiatric interviews by the Indonesian research team. PIU groups were included if they scored ≥ 45 on the IAT and were assessed through criteria previously outlined [46]. Control groups were selected if they scored < 45 on the IAT. All subjects had a past-year history of internet use, with the primary internet usage reported being 59.6% social media, 21.1% online games, 15.8% online forums/blogs, and 3.5% online music/video. Exclusion criteria included subjects with a history of any substance use, substance use disorder, or serious mental illness (as defined by the National Institute of Mental Health [47]). History of substance use was examined through Alcohol, Smoking, and Substance Use Involvement Screening and Testing (ASSIST) [48]. Recent substance use, or absence, was qualitatively confirmed through a urine dipstick test. Mini-International Neuropsychiatric Interview-Kid (MINI KID) [49] was used to screen for severe psychiatric disorders.

### 2.2. Psychological Questionnaires

#### 2.2.1. The Indonesian Version of Internet Addiction Test (IAT)

The IAT is a self-rating questionnaire used to measure internet addiction disorder [50]. The IAT had been validated into Bahasa Indonesia [51] and exhibited good psychometric properties comprised of 18 questions (Cronbach’s alpha 0.855). It employs a 6-point Likert scale (score 0 to 5) with total scores ranging from 0 to 90. Scores are classified as normal (score 0–27), mild PIU (score 28–44), moderate PIU (score 45–71), and severe PIU (score 72–90). In this study, PIU was determined with IAT score ≥ 45 (moderate to severe PIU).

#### 2.2.2. The Indonesian Version of Strength and Difficulties Questionnaire (SDQ)

The SDQ is a self-report questionnaire to measure emotional and behavioral problems. This questionnaire consists of 25 questions divided into 5 domains (emotional symptoms, conduct problems, hyperactivity problems, peers’ problems, and prosocial problems) and has been validated in Indonesian. Each question uses a 3-point Likert scale (0: not true, 1: somewhat true, 2: certainly true). Each domain score shows the problem’s magnitude of the particular domain. The score for each subscale is gained through summation of each item, thus generating a minimum score of 0 and a maximum of 10 for each subscale. The Indonesian version of the SDQ has good internal validity of Cronbach’s α = 0.773 [52].

#### 2.2.3. The Indonesian Version of Temperament and Character Inventory (TCI)

The Indonesian version of the modified TCI was used to measure temperament by employing only the first half of the instrument [53]. The questionnaire consists of 23 questions assessing the three domains of temperament, namely, NS, RD, and HA. The other 16 questions that assess character (self-directedness and self-transcendence) were not included in the present study. The confirmatory factor analysis demonstrated that the Indonesian version has a good model fit [54]. The answers were either “Yes” (2 points) or “No” (1 point), and the points were summed up according to each domain.

### 2.3. Functional Connectivity Analysis

Resting-State Functional Magnetic Resonance Imaging (rsfMRI) was performed to assess alteration of brain rsFC in adolescents with PIU compared to the control. The data were acquired by using an MRI Scanner (GE system 3.0 Tesla) with the T2 *-weighted gradient echo-planar imaging (EPI) sequence. The parameters used for the resting-state data were as follows: TR/TE 2000/30 ms, flip angle of 90 degrees, field of view (FOV) 25.6 × 19.2 cm, matrix size 64 × 64 mm, slice thickness 4 mm with 1 mm inter-slice gap and 30 slices. The examination was performed for 10 min with 300 total volumes. The recruitment and scans were completed between July 2018 and July 2019.

Image preprocessing was performed by the following procedures: first, FMRIB’s ICA-based X-noiseifier (FIX) was used for rsfMRI data to remove noises/artifacts such as head motion, heartbeat, respiratory fluctuation, and machine artifacts [55]. Second, the data were preprocessed using the CONN-fMRI FC toolbox (Connectivity Toolbox ver.17e, RRID:SCR_009550, [56]) on the statistical parametric mapping software package SPM12 (Statistical Parametric Mapping Software ver.12, Wellcome Trust Centre for Neuroimaging, University College London, UK, RRID:SCR_007037) running on MATLAB (ver.2017a, The MathWorks, Inc., USA). This included realignment, slice-timing correction, spatial normalization to the standard space (MNI 152, RRID:SCR_005281), scrubbing, smoothing by 8 mm full-width at half-maximum, regression of white matter, CSF, and head-motion-derived fluctuation by CompCor strategy [57]. Three subjects were excluded because they had head motion indicated by more than a 20% volume with a 0.5 mm difference of head position from adjacent volumes, two subjects from the PIU group, and one subject from the control group [58].

The ROIs were selected based on the independent component analysis of the Human Connectome Project provided by CONN (https://www.nitrc.org/projects/conn, accessed 25 July 2019), which included the (i) Salience Network (SN): Anterior Cingulate Cortex (ACC), left and right Anterior Insula (aIns), left and right Rostral Prefrontal Cortex (RPFC), and left and right Supra Marginal Gyrus (SMG); (ii) Default Mode Network (DMN): Medial Prefrontal Cortex (MPFC), left and right Lateral Parietal Cortex (LP), and Posterior Cingulate Cortex (PCC); (iii) Central Executive Network (CEN): left and right Lateral Prefrontal Cortex (LPFC), and left and right Parietal Posterior Cortex (PPC).

### 2.4. Statistical Analysis

The relationship of rsFC values between two ROIs were compared between the PIU and non-PIU groups using CONN. The statistical threshold was set at a false discovery rate (FDR, q-value) of less than 0.05. Age and sex were treated as covariates. Subsequent statistical analyses were performed in IBM (USA) SPSS version 27.0. Pearson’s correlation analysis with bootstrapping was performed between TCI, SDQ, and IAT scores. Interactions between IAT scores and diagnostic groups on the rsFC values of significant group differences were checked using MANOVA. If there was no interaction, we performed mediation analysis using PROCESS ([59], RRID:SCR_021369) to explore whether emotional and behavioral problems mediated the association between IAT scores and significant values of rsFC. Demographics, internet duration usage, and noncorrelated SDQ subscales were controlled for in the analysis. Nonparametric bootstrap was analyzed for the indirect pathway (PIU—Psychological Symptoms-rsFC) with 5000 samples and a 95% bias-corrected confidence interval. A significant mediation effect was considered if *p* < 0.05 and 95% CI did not cross zero.

## 3. Results

### 3.1. Demographic and Psychometric Data

The control and PIU group did not differ significantly in age, 13.9 ± 1.5 (51.7% in senior high school) versus 14.4 ± 1.6 (57.1% in senior high school), respectively. About 37.9% were male in the control group and 50% in the PIU group. On average, the IAT and emotional symptoms (SDQ subscale) scores for the PIU group and control group differed significantly. Detailed descriptive data are shown in Table 1.

### 3.2. Comparison of Resting-State Functional Connectivity between PIU and Control Groups

**Figure 1:**
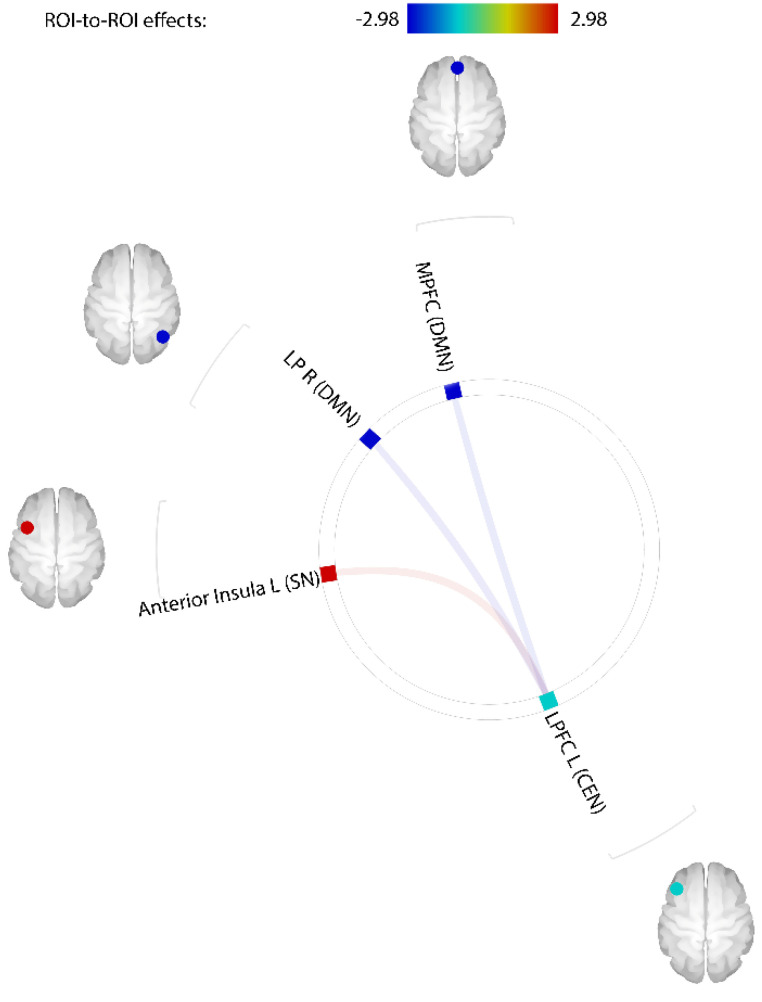
Comparisons of rsFC values between PIU group and control group. rsFCs of the PIU group, in comparison to control group, were higher between left LPFC and left AI (T (53) = 2.83, *q* = 0.044), but lower between left LPFC and MPFC (T (53) = −2.93, *q* = 0.044) and between left LPFC and right LP (T (53) = −2.98, *q* = 0.044). rsFCs, resting-state Functional Connectivity; PIU, Problematic Internet Use; LPFC, Lateral prefrontal cortex; MPFC, Medial prefrontal cortex; LP, Lateral parietal; aIns, Anterior insula.

Figure 1 displays the comparisons of rsFC between the PIU and control groups. The rsFC value was higher for PIU than for the control between the left LPFC and left anterior insula (T (53) = 2.83, *q* = 0.044). In contrast, rsFC values were significantly lower for the PIU group compared to the control between the left LPFC and MPFC (T (53) = −2.93, *q* = 0.044) and between the left LPFC and right LP (T (53) = −2.98, *q* = 0.044).

### 3.3. Associations between Problematic Internet Use Scores, Psychological Problems, and Temperament

IAT scores were demonstrated to have a moderate positive correlation with emotional symptoms (r = 0.38, *p* < 0.001) and conduct (r = 0.26, *p* < 0.05) problems, subscales of the SDQ. We found no correlation between scores of the IAT and all subscales’ scores of the TCI, as shown in Table 2.

### 3.4. Mediation Analyses of Functional Connectivity

**Figure 2:**
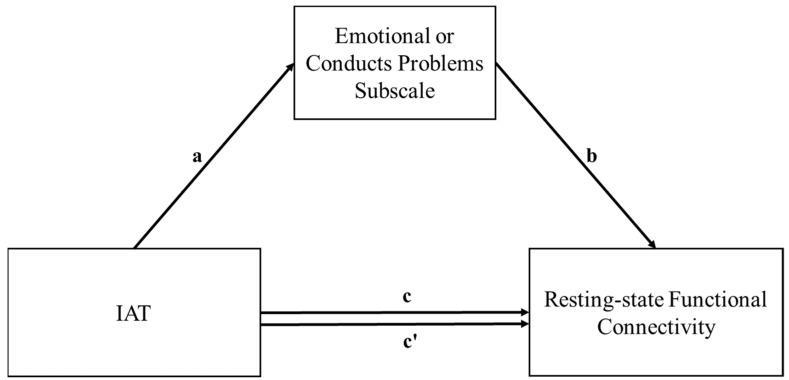
Conceptual diagram of mediation analysis between internet addiction, functional connectivity, emotional symptoms, and conduct problems subscales of SDQ. Resting-state functional connectivity includes: LPFC(L)-MPFC, LPFC(L)-LP(R), and LPFC(L)-aIns(L). a: independent variable direct effect on mediator; b: mediator direct effect on dependent variable; c: direct effect of independent on dependent variable; c’: indirect effect of independent on dependent variable, ab: indirect effect of independent on dependent variable through mediator. LPFC (L), Left lateral prefrontal cortex; MPFC, Medial prefrontal cortex; LP(R), Right lateral parietal; aIns(L), Left anterior insula.

There was no significant interaction between IAT scores and diagnosis (PIU or healthy control) to the rsFC of LPFC(L)-MPFC (*p* = 0.22), LPFC(L)-aIns(L) (*p* = 0.95), or LPFC(L)-LP(R) (*p* = 0.17), and the two groups were treated as having the same regression slopes. Emotional symptoms and conduct problems were further assessed through mediation analysis. The conceptual model of mediation is depicted in Figure 2. As shown in Table 3, IAT scores were found to associate positively, in a directed relationship, with scores of the emotional problem subscale of the SDQ (β = 0.054, *p* = 0.011). The indirect effect of the IAT on LPFC(L)-MPFC through emotional problems was significant (standardized β = −0.12, 95% CI −0.29, −0.0052). IAT scores did not predict conduct problem scores of the SDQ in a directed manner (β = 0.021, *p* = 0.18); thus, mediation analysis was not continued. Emotional symptoms did not mediate IAT scores to the other two rsFCs, LPFC(L)-aIns(L) and LPFC(L)-LP(R).

## 4. Discussion

The present study discovered a higher connectivity between the left LPFC (CEN) and left aIns (SN), among adolescents screened with PIU, but reduced connectivity of the left LPFC to both the right MPFC (DMN) and LP (DMN), in accordance with our hypothesis. Further, emotional problems mediated the relationship between IAT scores and between the LPFC(L) and MPFC (that is, between the CEN and DMN). In summary, we demonstrated that the rsFCs in the triple networks were altered in PIU and were partially mediated by emotional problems.

Compared to those within the normal range, subjects with problematic scores of the IAT had weaker rsFC values between the LPFC(L) to MPFC and LP(R), both of which are nodes within the DMN. The observed lower value was in line with the result of prior studies of addiction disorders [20,60]. The PFC can be classified into its medial and lateral parts, with the former comprising Brodmann areas (BA) of 9–12 and 25 and the latter BA 9–12 and 44–46 [61]. The MPFC has a significant role in self-referential judgment, social behavior judgment, and emotional and motivational regulation [62,63]. In contrast, the LPFC oversees motor control, performance monitoring (behavior rules and goals), and higher-order sensory processing [64]. The reciprocal connections between the LPFC and MPFC are vital for processing, controlling, and integrating affective valence and cognitive information, which determine decision-making and response inhibition [61].

The present study demonstrated that the rsFC between the LPFC(L) and MPFC was mediated through emotional problems in adolescents. Poor affective and emotional control has been postulated as a central node in adolescents’ psychopathologies [65], and the emotion-focused coping mechanism worsens PIU among adolescents [43]. Training in emotion regulation has been shown to effectively improve symptoms of substance use and gambling (behavioral addiction) disorder [66,67]. Thus, targeting adolescents’ adeptness to emotional states might help strengthen the response inhibition to addiction-related stimuli. The LPFC and LP(R) are essential hubs for memory-executive function processing by recalling the subjective experience of past events [68] and incorporating it as apparatus of self-referential or egocentric judgment [69]. Together, all these functions have been shown to be impaired in individuals with SUD and addictive behaviors, including PIU [70,71].

By contrast, PIU subjects demonstrate a higher connectivity between the LPFC(L) and aIns, which represent the connection between the CEN and SN. The dorsal LPFC, an integral component of the CEN, plays a role in the planning process, problem-solving, abstract thinking, attention, verbal function, and inhibition control. Adequate inhibition control allows individuals to limit inappropriate responses toward stimuli, including regulating individual involvement in reward-seeking behavior [72,73]. Individuals with PIU were found to experience inhibition control disruption resulting in difficulties in controlling urges and impulses to surf the internet [74,75], thus making it harder for them to control their internet use duration and preoccupation [76]. The aIns administers input of auditory, visual, behaviorally relevant, and self-referential stimuli and converge the signal with other regions, e.g., the amygdala and ventral striatum, to process the emotional and reward saliency of the stimuli. The LPFC and regions responsible for cognitive control in adolescents have delayed maturation compared to the aIns and other structures contributing to reward processing [77]. Thus, the bottom-up information, which is stimulus-driven and salient, is stronger than the top-down-inhibiting signals [77,78,79], explaining the greater rsFC in PIU. Indeed, subclinical internet use had also been found to correlate positively to nodes within the motivation/reward network, including the aIns [80].

Notably, the SN, CEN, and DMN components of ICNs have been implicated within a triple-network model of psychopathologies [28]. Studies have suggested that the SN (particularly the aIns) maintain a level of background activity to initiate brain responses toward salient stimuli, which could be overactive in psychopathologies, thus providing the basis of heightened salient detection in certain disorders [28]. Consequently, the SN plays a critical role in switching between the CEN and DMN engagement by shifting the attention from internal focus to an external one after detecting external stimuli [81]. The other two networks work to filter aberrant salience and allocation of attentional resources [28]. This involves the individual decision-making on whether to engage and maintain the ongoing behavior. Disruption in the SN was found to be related to persistent engagement in internet activities despite alternative cues of other activities [82]. In the context of PIU, the reduced function of the CEN leads to poor inhibition control, and the disruption occurring in the SN and DM resulted in inadequate internal control of preoccupation and an unrestrained urge and salience to utilize the internet [74,75,81].

The current study contained several limitations. First, the number of subjects was moderate, although comparable to other international studies [20,24,26,27]. Secondly, the analyses were cross-sectional, and we could not know about causation. The presented relationships might have alternative possibilities; for example, the emotional problems might also be the consequence of cognitive distortions in PIU apart from being the mediator. Further, the study was not designed to detail if the mediation became autonomous or required external emotional stimulation. Future studies should also incorporate active tasks to investigate the pathology of behavioral addiction. Nonetheless, to the best of the authors’ knowledge, this was the first study of functional cognitive analysis among screened PIU adolescents in Indonesia and employed a comparable proportion of male and female adolescents. Additionally, the study implemented stringent screening procedures to ensure that respondents did not suffer from other severely complicating psychopathologies and recruited test and control groups for analysis.

In conclusion, PIU adolescents exhibited increased connectivity between the nodes of the CEN and SN but decreased connectivity among the nodes of the CEN and DMN. This indicated the underlying neural mechanisms of poor cognitive and emotional response inhibition (DMN-CEN). Such altered connectivity was mediated by emotional problems, indicating a potential new intervention target.

## Figures and Tables

**Table 1:** Subjects’ demographic and descriptive scores.

Variables	Control (*n* = 29) ^a^	PIU (*n* = 28) ^a^	T/χ^2^
Age	13.9 ± 1.5	14.4 ± 1.6	−1.061
Sex (male/female) ^b^	11/18	14/14	0.843
Weekly Internet Duration	31.6 ± 15.1	41.1 ± 31.9	−1.444
IAT	27.2 ± 5.5	50.8 ± 6.1	−15.389 ***
Emotional Symptoms	4.1 ± 2.1	5.9 ± 2.2	−3.108 **
Conduct Problems	2.8 ± 1.4	3.5 ± 1.6	−1.656
Hyperactivity Symptoms	3.9 ± 1.6	4.5 ± 2.0	−1.262
Peer Problems	2.7 ± 1.2	3.0 ± 1.9	−7.33
Prosocial Behaviours	7.9 ± 2.2	7.1 ± 2.1	1.367
NS	11.1 ± 1.6	11.8 ± 1.7	−1.682
HA	9.9 ± 1.2	9.1 ± 1.5	2.045 *
RD	12.1 ± 1.2	12.4 ± 1.7	−0.57

Note: ^a^ Data presented as Mean ± SD; ^b^ Data presented as ratio and analyzed using *T*-test or chi-square; PIU, Problematic Internet Use; IAT, Internet Addiction Test; NS, Novelty Seeking; HA, Harm Avoidance; RD, Reward Dependence. * *p* ≤ 0.05; ** *p* ≤ 0.01; *** *p* ≤ 0.001.

**Table 2:** Correlations of IAT scores to SDQ and TCI subscales.

Variables	Correlation with IAT (Pearson’s r)
SDQ	
1. Emotional Symptoms	0.377 **
2. Conduct Problems	0.263 *
3. Hyperactivity Symptoms	0.198
4. Peer Problems	0.164
5. Prosocial Behavior	−0.168
TCI	
6. NS	0.289 *
7. HA	−0.143
8. RD	0.062

Note: IAT, Internet Addiction Test; SDQ, Strength and Difficulty Questionnaire; TCI, Temperament and Character Inventory; NS, Novelty Seeking; HA, Harm Avoidance; RD, Reward Dependence; *, *p* < 0.05; **, *p* < 0.001; bold values have significant 95% CI (does not cross zero) with 5000 bootstrap samples.

**Table 3:** Mediation analysis of SDQ subscales toward correlation of IAT scores and functional connectivity.

rsFC	Effect of IAT to Emotional Symptoms Subscale (a)		Direct Effect of Emotional Symptoms Subscale (b)		Direct Effect of IAT (c’)		Total Effect of IAT (C)		Indirect Effect of IAT (ab)		
	β	SE	β	SE	β	SE	β	SE	β	SE	Boot 95%CI
LPFC(L)-aIns(L)	0.054 **	0.02	0.097	0.017	0.0064 **	0.0025	0.007 ***	0.0024	0.0005	0.001	−0.0016, 0.0024
LPFC(L)-MPFC	0.054 **	0.02	−0.036 **	0.015	−0.0026	0.0023	−0.0045 *	0.0022	−0.002	0.0013	(−0.0051, −0.0001) ^+^
									**−0.12**	**0.074**	**(−0.29, −0.0052) ^‡^**
LPFC(L)-LP(R)	0.054 **	0.02	−0.0059	0.015	−0.0046 *	0.0023	−0.0050 *	0.0021	−0.0003	0.001	−0.0021, 0.0019

Notes: * *p* ≤ 0.05; ** *p* ≤ 0.01; *** *p* ≤ 0.001; ^+^ Unstandardized; ^‡^ Completely Standardized; rsFC, Resting-state functional connectivity; IAT, Internet addiction test; LPFC(L), Left lateral prefrontal cortex; aIns(L), Left anterior insula; MPFC, Medial prefrontal cortex; LP(R), Right lateral parietal cortex. Significant standardized beta coefficient of indirect effect is bolded.

## Data Availability

The data presented in this study are available on request from the corresponding author.
